# Effect of Cold Acclimation on Selected Metabolic Enzymes During Diapause in The European Corn Borer *Ostrinia nubilalis* (Hbn.)

**DOI:** 10.1038/s41598-020-65926-w

**Published:** 2020-06-03

**Authors:** Iva Uzelac, Miloš Avramov, Tatjana Čelić, Elvira Vukašinović, Snežana Gošić-Dondo, Jelena Purać, Danijela Kojić, Duško Blagojević, Željko D. Popović

**Affiliations:** 1 University of Novi Sad, Faculty of Sciences, Department of Biology and Ecology, Trg Dositeja Obradovića 2, 21000 Novi Sad, Serbia; 2 Maize Research Institute, Zemun Polje, Slobodana Bajića 1, 11185 Belgrade, Serbia; 3 Institute for Biological Research “Siniša Stanković”, Bulevar despota Stefana 142, 11060 Belgrade, Serbia

**Keywords:** Enzymes, Animal physiology

## Abstract

The European corn borer, *Ostrinia nubilalis* Hbn., is a pest Lepidopteran species whose larvae overwinter by entering diapause, gradually becoming cold-hardy. To investigate metabolic changes during cold hardening, activities of four metabolic enzymes – citrate synthase (CS), lactate dehydrogenase (LDH), alanine aminotransferase (ALT) and aspartate aminotransferase (AST) were measured in whole-body homogenates of pupae, non-diapausing and diapausing larvae acclimated to 5 °C, −3 °C and −16 °C. The highest CS activity was detected in non-diapausing larvae, reflecting active development, while the highest *in vitro* LDH activity was recorded in diapausing larvae at temperatures close to 0 °C, evidencing a metabolic switch towards anaerobic metabolism. However, in-gel LDH activity showed that production of pyruvate from lactate is triggered by sub-zero temperatures. The activities of both aminotransferases were highest in non-diapausing larvae. Our findings suggest that during diapause and cold hardening the aminotransferases catalyse production of L-alanine, an important cryoprotectant, and L-aspartate, which is closely tied to both transamination reactions and Krebs cycle. The results of this study indicate that, during diapause, the activity of metabolic enzymes is synchronized with exogenous factors, such as temperatures close to 0 °C. These findings support the notion that diapause is metabolically plastic and vibrant, rather than simply a passive, resting state.

## Introduction

Diapause, a period of temporarily arrested development in the life cycle of numerous species inhabiting temperate and polar zones, is an adaptation by which organisms synchronize their activities with seasonal changes. Since it is induced ahead of the harsh environmental conditions, it enables organisms to successfully cope with numerous stressful factors, such as low winter temperatures, limited or unavailable food and water sources, etc. A complex phenomenon, diapause consists of three major phases – pre-diapause, diapause and post-diapause (quiescence), giving the diapausing organism time to gradually change^1^. Plenty of biochemical and molecular adaptations occur during the different phases of diapause, including: metabolic depression^2^; reduction in the activity of low-priority metabolic pathways and simultaneous redirection of metabolism towards the synthesis of specific cryoprotective molecules^3–8^; changes in the fatty acid composition of membrane and storage lipids^9–11^, and expression of specific sets of genes and proteins which are involved in stress protection and regulation of cell cycle and programmed cell death^12–16^. In addition, prior to diapause entry, an organism is subjected to different behavioural activities such as migration, locating suitable micro-habitats, aggregation, and physiological processes such as accumulation of energy reserves, which altogether lead to increased stress tolerance^1^.

The European corn borer (ECB), *Ostrinia nubilalis* (Hübner), is a moth species native to Eurasia, however today widely spread across North America and northern Africa. Larvae of this species have an extremely wide range of more than 200 host species, including tomatoes, potatoes, snap beans, peppers, sorghum, corn, and many weed species^17^. The ECB is a holometabolous insect whose overwintering larvae enter diapause during the fifth (final) instar stage and gradually become cold hardy^18,19^. Diapause is an adaptive strategy for surviving harsh winter conditions and it is inextricably linked with the development of cold hardiness. Biochemical and molecular adaptations in diapause involve the synthesis of cryoprotectors such as polyhydroxyl alcohols (glycerol, sorbitol, etc.) and carbohydrates (trehalose, glucose, etc.), which colligatively decrease the haemolymph’s supercooling point while, at the same time, non-colligatively stabilize proteins and cell membranes^3,8,14,20–23^. In accordance with that, in the previous GC-MS and ^1^H-NMR based metabolomic studies of this species, it was established that the haemolymph of actively developing and diapausing larvae has completely distinct metabolomic profiles^8,24^. Namely, the haemolymph of non-diapausing larvae is enriched in lactate, acetate and succinate, which indicates intensive catabolism to meet the high energy demands for active development. On the other hand, natural cryoprotectants such as glycerol, proline and alanine, are the predominant metabolites in the haemolymph of diapausing and cold acclimated diapausing larvae^3,8,23,24^.

In order to investigate whether the relationship between carbohydrate and amino acid metabolisms reflects the potential shift from aerobic to anaerobic metabolism during distinct developmental phases of *O. nubilalis*, the activities of four metabolic enzymes – citrate synthase (CS), lactate dehydrogenase (LDH), alanine aminotransferase (ALT) and aspartate aminotransferase (AST) were assessed in whole-body homogenates of pupae (P), non-diapausing (ND) and diapausing larvae (D). Furthermore, because of a close connection between cold hardiness and diapause in the ECB^3,20,21,25,26^, diapausing larvae were acclimated to different low temperatures (5 °C, −3 °C, and −16 °C), in order to track the potential effect of chilling on the activities of the selected enzymes.

Citrate synthase (CS, EC 2.3.3.1) is an essential component of the Krebs cycle^27^ and is present in almost all known living organisms. It catalyses the condensation reaction of a four-carbon molecule, oxaloacetate, and the two-carbon acetate residue from acetyl coenzyme A, to form the six-carbon citrate. CS is a marker of oxidative metabolism and is often used to investigate specific cellular adaptations of organisms living in hypothermic and hypoxic environments^28–30^.

Lactate dehydrogenase (LDH, EC 1.1.1.27) catalyses the interconversion of lactate and pyruvic acid, with the simultaneous oxidoreduction of the nicotinamide adenine dinucleotide (NAD^+^/NADH) coenzyme. It is the terminal enzyme of anaerobic glycolysis in the cytosol and, by producing lactate and regenerating NAD^+^, it facilitates continued ATP production by glycolysis^31^. A high level of LDH activity is associated with an effective anaerobic system and insect tissues with predominantly anaerobic metabolism are enriched with this enzyme, in particular muscles characterized by rapid and convulsive activity^32^.

Alanine aminotransferase (ALT, EC 2.6.1.2) catalyses the reversible reaction of transamination between L-alanine and α-ketoglutarate. ALT transforms alanine into pyruvate and keto acids into amino acids. In this way, via pyruvate and glutamate, ALT bridges carbohydrate and amino acid metabolisms^33^. ALT is also involved in the *de novo* biosynthesis of alanine, which is one of the most common natural cryoprotectants and precursors for gluconeogenesis^34–36^.

Aspartate aminotransferase (AST, EC 2.6.1.1) catalyses the reversible reaction of transamination between L-aspartate and α-ketoglutarate. Similarly to ALT, AST also brings together the catabolism of amino-acids and carbohydrates, as well as connects cytosolic and mitochondrial compartments via the malate-aspartate shuttle. In insects, high AST activity is detected in the fat body which is probably the main site of amino acid interconversion^37^, analogous to the liver in vertebrates.

Aerobic metabolism is a common characteristic of active life, while anaerobic metabolism is preferred in dormant states and thus we presumed that the activities of CS (an aerobic marker) and LDH (an anaerobic marker) should be contrasted in diapausing and non-diapausing larvae. This is supported by the fact that the reduction in CS activity was already observed in the diapause of tropical^38^, subpolar and continental insects^39,40^, as well as in the dormant phase of a calanoid copepod *Calanus glacialis*^41^. Besides, there is evidence that a decreased metabolism rate is accompanied by reduced relative abundance of CS coding gene transcripts during reproductive diapause in a heteropteran bug *Pyrrhocoris apterus*^42^. On the other hand, previous results concerning LDH activity and lactate content are not as consistent. For example, larvae of the goldenrod gall moth *Epiblema scudderiana* have a lower metabolism rate during winter, but they do not accumulate lactate^43^, unlike larvae of the goldenrod gall fly *Eurosta solidaginis* which enter an anaerobic state due to freezing and consequently accumulate lactate^44^. Analysis of LDH activity on starch-gel electrophoresis in the pink bollworm *Pectinophora gossypiella* revealed a higher activity in non-diapausing larvae^45^, perhaps due to the rearrangement of different LDH subunits during diapause. Low temperature exposure could also differently affect LDH in different tissues of the eri silkworm *Philosamia ricini* – LDH activity showed a significant decrease in the haemolymph and increase in the fat body^46^.

As for aminotransferases, it was expected in this study that their activities in the direction of pyruvate and oxaloacetate synthesis would be higher in non-diapausing compared to diapausing larvae, due to increased metabolic rate during active development. A previous study on the solitary bee *Osmia rufa* showed that activity of aminotransferases could also be gender-dependent. Namely, activities of both ALT and AST were higher in the haemolymph of non-diapausing compared to diapausing females, while males were unresponsive to diapausing conditions^47^. However, completely opposite findings were observed in the fat bodies – both ALT and AST activity were significantly decreased in non-diapausing compared to diapausing bees of both genders^47^. In addition, exposure to low temperatures also influences activities of these enzymes – both ALT and AST were significantly inhibited in the haemolymph, while their activity showed a very drastic increase in the silk gland of the eri silkworm *Philosamia ricini* after cold acclimation^48^.

 The role of these enzymes during diapause in the European corn borer has not yet been investigated, giving this study special significance in understanding the physiological strategy adopted by this economically important species to withstand harsh winter conditions.

## Results

**Figure 1:**
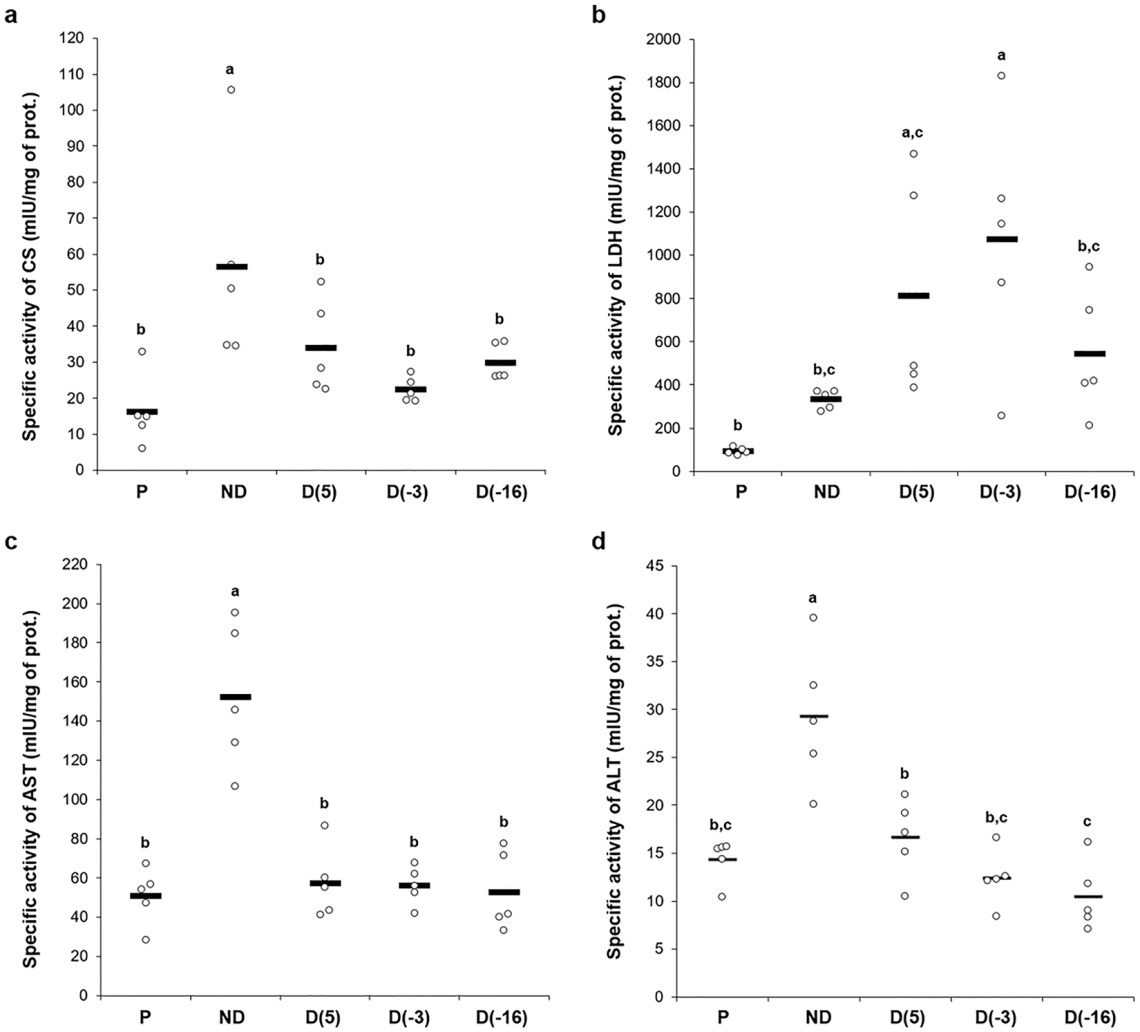
Specific enzyme activities of: citrate synthase (**a**), lactate dehydrogenase (in the direction from pyruvate to lactate) (**b**), aspartate aminotrasferase (in the direction from L-aspartate to oxaloacetate) (**c**) and alanine aminotransferase (in the direction from L-alanine to pyruvate) (**d**). All results are expressed in mIU/mg of prot. and presented as univariate scatterplots, where each dot represents one biological pool comprised of 10 larvae or pupae. The statistical significance of determined values was tested using one-way ANOVA followed by *post hoc* Fisher’s test, with a level of significance of p < 0,05. Statistically significant results are labeled with different letters above the scatterplots.

The results of CS, LDH, ALT and AST activities, measured in whole-body homogenates of pupae, non-diapausing and cold-acclimated diapausing larvae, are shown in Fig. 1. In general, the activities of CS, AST and ALT were the highest in non-diapausing larvae, compared to diapausing larvae and pupae, while the activity of LDH was the highest in diapausing larvae acclimated to −3 °C (Fig. 1). On the other hand, the lowest activities of enzymes were recorded either in pupae (CS and LDH) or in the −16 °C diapausing group (ALT) (Fig. 1). More precisely, CS displayed around a 2-fold increase in non-diapausing relative to diapausing larvae and a 4-fold increase relative to pupae, while LDH activity was 3-fold higher in diapausing larvae acclimated to −3 °C in comparison to non-diapausing larvae and even 10-fold higher in comparison to pupae. Relative to all other groups, AST activity was 3-fold higher in non-diapausing larvae, whereas ALT activity showed around 2-fold increase in non-diapausing larvae in comparison to pupae and the D(5) group, and 3-fold increase relative to the D(−3) and D(−16) groups.

Enzyme activities between the diapausing and non-diapausing larvae of *O. nubilalis* were significantly different for all analyzed enzymes (Fig. 1; One-way ANOVA, p < 0,05). However, exposure of diapausing larvae to low temperatures did not have a consistent impact on the activities of all selected enzymes. The activities of CS and AST were not responsive to low temperature treatment, in contrast to LDH and ALT. Exposure to temperatures close to 0 °C has led to a significant increase of LDH activity in diapausing larvae of D(5) and D(−3) groups. Also, a gradual but significant decrease of ALT activity has been recorded during the chilling of diapausing larvae, with the lowest value in the D(−16) group (Fig. 1).

**Figure 2:**
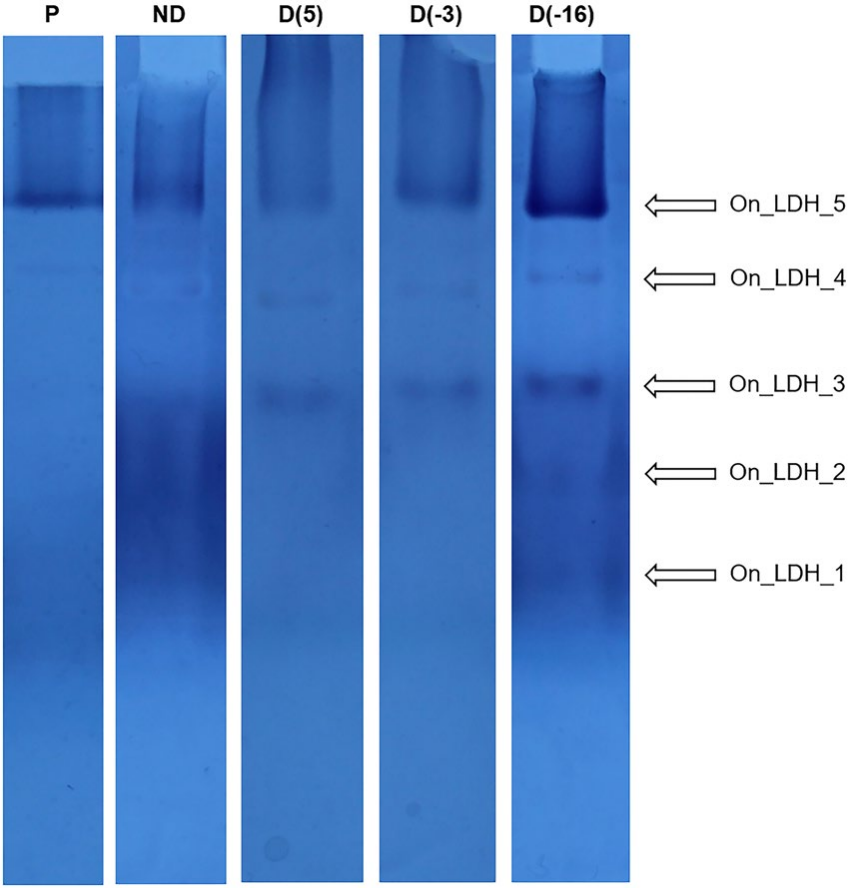
Electrophoretic pattern of lactate dehydrogenase isoenzymes from whole-body homogenates (30 μg of total proteins per well) of: P−pupae; ND−non-diapause; D(5), diapausing larvae acclimated at 5 °C; D(−3), diapausing larvae acclimated at −3 °C and D(−16) diapausing larvae acclimated at −16 °C. The gel photograph is representative of experiments performed in five biological pools (n = 10 larvae/pupae per pool). Individual lanes have been cropped and grouped from different gels representing each biological pool. Full-length gels are available in the Supplementary material.

**Figure 3:**
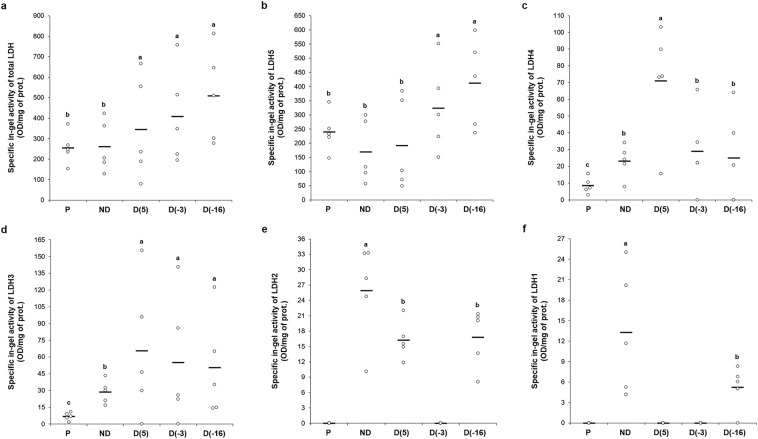
Specific in-gel measured activity of total LDH (**a**) and individual detected isoforms − LDH5 (**b**), LDH4 (**c**), LDH3 (**d**), LDH2 (**e**) and LDH1 (**f**); in the direction from lactate to pyruvate. All results are expressed in OD/mg of prot. obtained from the optical density (OD) analysis of Supplementary material performed in ImageJ and presented as univariate scatterplots, where each dot represents one biological pool. Total LDH activity is the sum of individual isoform activities detected in a biological pool. The statistical significance of determined values was tested using one-way ANOVA followed by *post hoc* Fisher’s test, with a level of significance of p < 0,05. Statistically significant results are labeled with different letters above the scatterplots.

In addition, since the spectrophotometric LDH assay measures the activity of LDH in the direction of lactate and NAD^+^ production, we decided to determine whether the activity of LDH was altered in the opposite direction – towards the production of pyruvate and NADH. Thus, the activity of LDH, in the direction from lactate to pyruvate, was assayed in-gel after native PAGE of whole-body homogenates and five LDH isoforms (On_LDH_1-5) were detected with NBT test (Figs. 2, 3). The size i.e. relative abundance of the bands was quantified in the program ImageJ, in order to compare enzyme activity of various LDH isoforms among the different treatments, and the results are presented in Fig. 3. Isoforms On_LDH_3-5 were present in all experimental groups, On_LDH_2 in D(5), D(−16) and ND groups, while On_LDH_1 was clearly visible only in the D(−16) and ND groups. The activity of LDH isoforms corresponds to the intensity of detected bands. It can be noted that the total LDH activity was higher in diapausing groups, especially in the D(−16) group, in comparison to non-diapausing larvae and pupae, which had similar total in-gel activity (Figs. 2, 3a).

**Figure 4:**
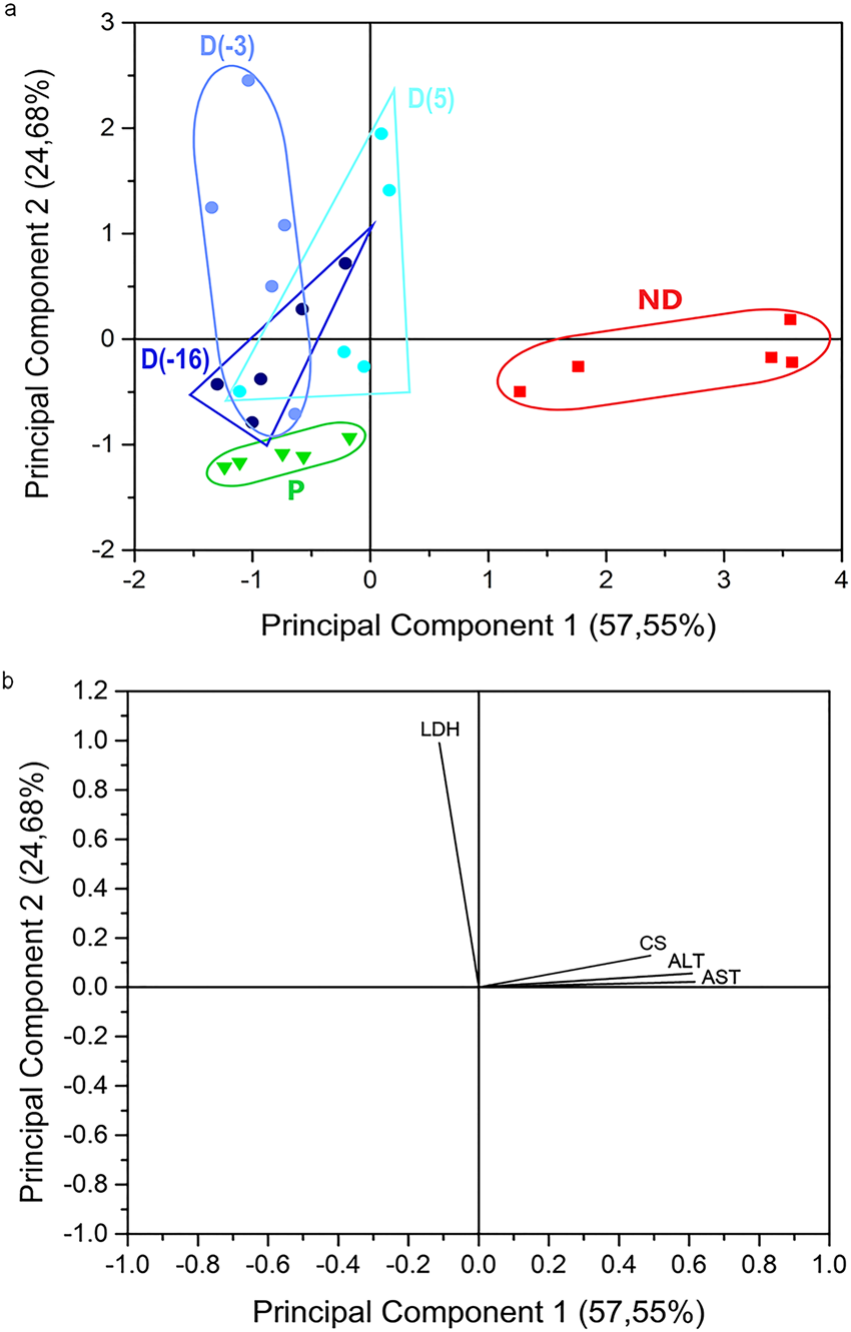
Plots of component scores (symbols) (**a**) and of the variable loadings (vectors) (**b**) for the two principal components from PCA performed on the activities of CS, LDH, ALT and AST: P−pupae; ND−non-diapause; D(5), diapausing larvae acclimated at 5 °C; D(−3), diapausing larvae acclimated at −3 °C and D(−16) diapausing larvae acclimated at −16 °C. Scores are scaled by the square root of the eigenvalues (i.e., scaling = 2), and all samples consisted of five independent groups (10 larvae/pupae per group).

In order to reveal potential links between patterns of enzyme activities, developmental stage and cold acclimation, we subjected data from specific enzyme activities to principal component analysis (PCA) and presented these results in Fig. 4. A PCA score plot (Fig. 4a) shows clusters of samples based on their similarity, while a PCA loading plot (Fig. 4b) shows how strongly each characteristic influences a principal component. PCA captures the essence of the data in a few principal components, which convey the most variation in the dataset. As we can see in Fig. 4, the first two principal components extracted by PCA covered 82.23% of the variance in the data. The first principal component (PC1) accounted for 57.55% of the total variance, and it is highly likely that this dimension separated samples according to developmental stage (Fig. 4a) since PC1 separated ND (positive scores) from P and the D groups (mostly negative scores). Moreover, PC1 was positively correlated with the activities of all enzymes, except for LDH which had shown weak negative correlation (Fig. 4b). The second principal component (PC2), although less substantial, accounted for 24.68% of the total variance. PC2 to some extent reflects the effect of cold acclimation on enzyme activities in diapausing larvae, since it mostly separated the −3 °C diapausing group from the other two cold-acclimated groups (Fig. 4a). PC2 had a strong positive correlation with LDH activity and a weak positive correlation with the activities of all other enzymes (Fig. 4b).

## Disscusion

One of the hallmarks of hypometabolism is the shift from aerobic to anaerobic metabolism, which is inextricably connected with various types of dormant states of organisms^49,50^. The enzymes which could be potential markers of the intensity of aerobic processes in a given organism, such as citrate synthase, aspartate aminotransferase and alanine aminotransferase, should be particularly affected by this shift, since their products are directly or indirectly involved in the Krebs cycle. Considering that the Krebs cycle is a key metabolic pathway for releasing energy stored in carbohydrates, lipids and proteins, its intensity is often at lower levels during diapause, as a result of a general suppression of aerobic metabolism. Previous studies on *O. nubilalis* have reported lower rates of oxidative metabolism during diapause^4,5,18^. Similar findings were reported in other insects that enter diapause^51,52^. On the other hand, in actively developing *O. nubilalis* larvae, higher concentrations of acetate, succinate and glutamine – indicators of intense oxidative metabolism of lipids and carbohydrates, were detected in our previous metabolomic study^24^.

In the present study, the highest activities of CS, AST and ALT were recorded in non-diapausing, metabolically highly active larvae, while their activities were considerably lower in pupae and cold acclimated diapausing larvae. The activity of LDH, whether assayed *in vitro* (Fig. 1b) or in-gel (Fig. 3a), was higher in diapausing in comparison to non-diapausing larvae and pupae. In pupae, the activities of CS and LDH were lower in comparison to all other experimental groups, while the activities of AST and ALT were mostly at similar levels to those in diapausing larvae. Such findings reflect the altered pupal metabolism and can be explained by the concurrent histolysis of larval and histogenesis of adult tissues, two metabolically opposed processes. Similarly to our findings, low CS activity was observed in larvae of two insect species, *Eurosta solidaginis* (Diptera) and *Epiblema scudderiana* (Lepidoptera)^39^, in diapausing pupae of the flesh fly *Sarcophaga crassipalpis*^40^ and the dormant phase of the calanoid copepod *Calanus glacialis*^41^. Moreover, in *C. glacialis*, both citrate synthase and malate dehydrogenase activities decrease significantly from July to December, suggesting that basic metabolic rates were reduced during overwintering^41^.

**Figure 5:**
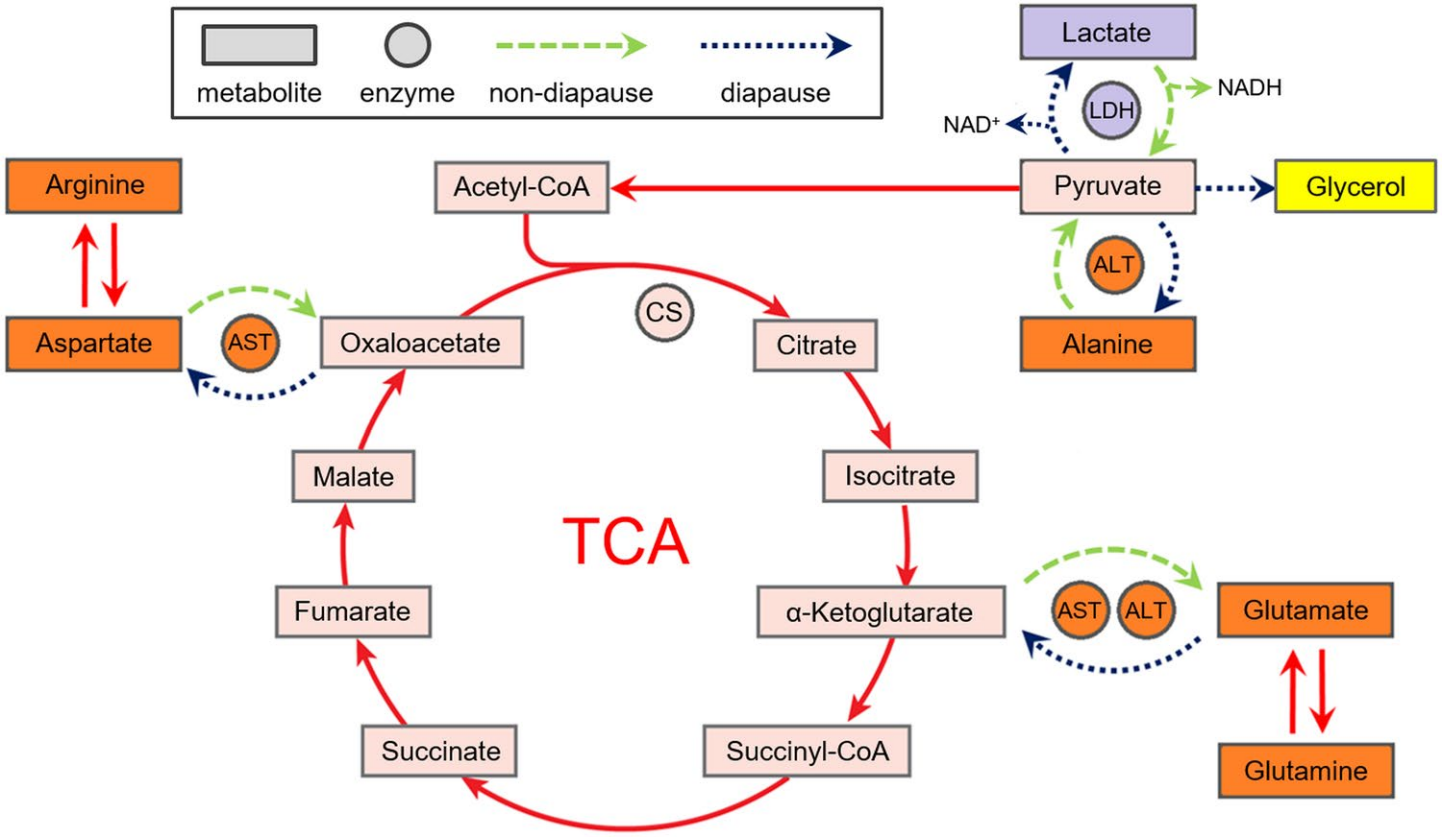
The proposed metabolic pathways during the active life and diapause in *Ostrinia nubilalis* (explanation given in the text). TCA – tricarboxylic acid (Krebs) cycle, ALT – alanine aminotrasferase, AST – aspartate aminotrasferase, CS – citrate synthase, LDH – lactate dehydrogenase.

Since the substrates/products of transaminases are closely tied to the Krebs cycle (Fig. 5), it is expected for their activities to be altered during diapause and cold hardening. In this study, AST and ALT activities were assayed towards the conversion of L-aspartate into oxaloacetate and L-alanine into pyruvate, respectively. Both AST and ALT were shown to be highly active in non-diapausing larvae in comparison to pupae and diapausing larvae, which is in accordance with the high intensity of metabolism in the actively developing larvae. Furthermore, a cooling treatment during diapause affected the activity of ALT, which gradually decreased along with the temperature, while the activitiy of AST remained unchanged (Fig. 1c,d).

Likewise, in the study of cold-stressed fifth instar larvae of the silkworm *Philosomia ricini*, it has been shown that activities of both ALT and AST were significantly inhibited in the haemolymph after exposure to low temperatures, in comparison to the control group reared at ambient temperature^48^. Although this Lepidopteran species does not enter diapause, its larvae also accumulate high levels of glycerol in the haemolymph, silk gland and fat body, in response to cold stress^48^, similar to the ECB. Thus, reduced activities of transaminases in the haemolymph might be a consequence of a general metabolic suppression during cold stress response in insects. However, it is interesting that, at the same time and in the same conditions, the activities of transaminases showed a very drastic increase in the silk gland of the eri silkworm^48^, probably due to the fact that in the final larval instar (fifth instar in *Philosomia ricini*) the silk glands develop according to a metamorphic pattern that differs from the larval one by enhanced function and, especially, by the programming of silk glands for histolysis^53^.

Opposite to CS, activity of LDH could be a good indicator of anaerobic metabolism, since this enzyme catalyses the conversion of pyruvate into lactate in hypoxic and anaerobic conditions. In the present study the activity of LDH was spectrophotometrically measured in the direction towards lactate production and assayed in-gel in the opposite direction, towards pyruvate production. LDH activity in the direction of lactate production was lower in non-diapausing larvae, compared to diapausing, cold-acclimated larvae (Fig. 1b). However, these findings might be unexpected since the increased amount of lactate was previously recorded in the haemolymph of actively developing larvae^24^. This could be explained with the fact that high amounts of lactate are toxic and therefore this metabolite might be excreted into the haemolymph, rather than stored within the cells. Then, during prolonged hypometabolism in diapause, other tissues with high energy demands may reabsorb lactate from the haemolymph via lactate/pyruvate transporters and reconvert it into pyruvate, in a similar manner as recently described in *Drosophila* brain under starvation stress^54^. An alternative explanation could be that high amounts of lactate in the haemolymph of non-diapausing larvae might inhibit further production of lactate in other tissues via a negative feedback loop on the LDH and thus the production of pyruvate in an energy-limited state of diapause and cold acclimation is favoured, as was confirmed in this study by in-gel assay of LDH activity (Figs. 2, 3).

The results from the in-gel activity assay revealed the presence of five different LDH isoforms - On_LDH1-5 (Fig. 2), whose activities were apparently influenced by developmental stage, active or diapausing state and cold acclimation (Fig. 3b–f). Three isoforms, On_LDH3-5, displayed the highest activity in diapausing larvae **(**Fig. 3b–d**)**, while in pupae and non-diapausing larvae they had either a lower or a non-detectable in-gel activity. The remaining two isoforms, On_LDH1-2, had higher activity in the ND group compared to the rest (Fig. 3e,f). Nevertheless, the total in-gel measured LDH activity is evidently the highest in the diapausing larvae (Fig. 3a), as their acclimation to subzero temperatures lead to a further increase in the activity of most of LDH isoforms (Fig. 3b–d). It is well-known that lactate-to-pyruvate conversion, as confirmed with the in-gel activity assay in this study, serves to regenerate the reduced form of the coenzyme NADH. Thus, the reduced form of NADH could then be used as a source of reducing equivalents needed either in ATP production or in regeneration of small antioxidants within the antioxidative defense system (ADS) that has been well described in different developmental stages and temperature acclimations of *O. nubilalis*^4,5,7,15,55^. Since pyruvate is a reactive metabolite, not so suitable for a long-term storage, it is probably transformed into either glycerol or L-alanine, as shown in Fig. 5. Alanine is a fundamental amino acid, a non-toxic compound and at the same time might act as a cryoprotectant^56,57^ and thus, it could be a more favourable metabolic form for storing excess pyruvate, in comparison to the toxic lactate.

As for the diapausing larvae of the ECB, they acquire cold hardiness in response to the gradual decrease of temperatures around 0 °C by accumulating several major protective compounds – glycerol, trehalose, glucose as well as L-alanine and proline^8,23,24^. Therefore, in *O. nubilalis*, the activities of transaminases and LDH must be discussed in the context of both diapause and cold hardiness. As shown in this study, after cold acclimation of diapausing larvae, LDH activity increased significantly (up to 70%) and transaminase activities decreased (2 to 3-fold) compared to non-diapausing larvae (Fig. 1). Also, it has been reported that the levels of L-alanine and glycerol in the haemolymph of diapausing ECB larvae also significantly increased with cooling to −3 °C^24^.

Although the content of free amino acids in cold hardy insects is usually not as high as that of sugars and polyols^6,8,58^, these biomolecules also enrich the milieu of molecular cryoprotectants. Numerous studies on insects have shown that the levels of alanine and proline increase in response to low temperatures^34–36,44,58–62^ and that a proline-augmented diet could even improve survival of diapause-destined larvae exposed to liquid nitrogen^63^. Still, it is unclear whether alanine elevation is a by-product of low temperatures, in which case this elevation may be the side result of low-temperature inhibition or activation of an enzyme immediately upstream or downstream of alanine^40^, or alanine elevation plays a direct role in low-temperature survival, by serving as an alternative end-product to lactic acid when the downstream respiratory pathway is slowed or ceases to function^40^.

In the present study, ALT activity was measured in the direction from L-alanine to pyruvate and was lower in cold-acclimated diapausing larvae in comparison to non-diapausing larvae (Fig. 1). Since increased levels of L-alanine were previously found in the haemolymph of cold-hardy ECB larvae^24^, it is suggested that this excess of alanine could be the consequence of intense ALT activity in the opposite direction to the one measured in this paper – towards L-alanine production from pyruvate. However, a detailed study on cold-acclimated diapausing larvae is required to test this presumption before reaching any definitive conclusion. A limiting factor is the impossibility to couple the basic ALT reaction in the direction towards L-alanine production with the corresponding indicator reaction, which should be used to quantify the amount of produced L-alanine and, indirectly, ALT activity.

Analogous to ALT, lower AST activity in cold-acclimated diapausing larvae could mean that this enzyme is also more active in the opposite direction, towards the production of L-aspartate from oxaloacetate. Considering that the activity of CS and the intensity of the Krebs cycle are attenuated during diapause, excess reactive oxaloacetate is then probably redirected to synthesis of the less reactive L-aspartate (Fig. 5). Just like ALT, additional experiments are needed to distinguish in which direction AST is acting during diapause in the ECB. There have been only a few studies which investigated the link between diapause/cold hardiness and levels of L-aspartate, but the results were species-specific – aspartate levels were elevated due to cold acclimation in the flat grain beetle *Cryptolestes ferrugineus*^61^, but reduced in freezing frogs^64^ and snails^65^. Since none of the aforementioned studies employed transcriptomic or proteomic methods to confirm amino acid content alteration, Michaud and Denlinger^58^ suggested that these changes were probably the consequence of enzyme kinetics regulation, rather than gene induction.

Despite the limitations of the experimental design of this study, which prevents us from clearly separating the effects of cold acclimation and the diapause phenotype, our findings indicate that AST is probably more influenced by the endogenous diapause programme, rather than cold acclimation. In contrast to this, the activities of LDH, and to some extent CS and ALT, seem to be more influenced by the exposure to low temperatures, especially those around 0 °C. Moreover, a decrease in both ALT and *in vitro* LDH activities in diapausing larvae cooled to −16 °C, compared to those acclimated to 5 °C and −3 °C, suggests that fine tuning of metabolism occurs at temperatures close to zero, while a general metabolic suppression occurs at extremely low temperatures during ECB diapause, as confirmed by the in-gel LDH activity assay. Similar findings were described in diapausing larvae of the gall fly *Eurosta solidaginis*, where glycolysis stops at subzero temperatures and metabolites are also redirected towards the synthesis of sorbitol and other cryoprotective polyols^66^.

To our knowledge this is the first time that LDH isoforms have been detected in larvae and pupae of *O. nubilalis*. These results have showed that their activities substantially differ between diapause and actively developing states. Therefore, future experiments should characterise individual metabolites, measure differential gene expression and assess enzyme activity.

Superficially, diapause might seem like a passive state of developmental arrest. However, the results of this study, together with previous research on the ECB and other diapausing insects, demonstrate that the activity of metabolic enzymes is maintained in a state of dynamic equilibrium during diapause and is synchronized with exogenous factors, such as the onset of low temperatures. In this way, an organism shapes its metabolism according to the changes in the environment and directs it towards the synthesis of protective compounds (glycerol, alanine, etc.), on the one hand, and the concurrent removal of potentially harmful metabolites (lactate, pyruvate, etc.), on the other.

## Methods

### Insect collection and cold acclimation

**Figure 6:**
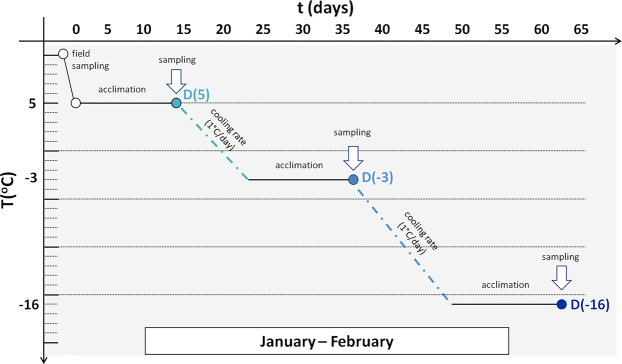
Cold acclimation experiment (explanation provided in the text). Diapausing larvae: D(5) – acclimated to 5 °C, D(−3) – acclimated to −3 °C and D(−16) – acclimated to −16 °C.

Diapausing (winter) and non-diapausing (summer) larvae of the ECB, as well as pupae, were sampled from the fields of the Maize Research Institute in Zemun Polje (44°87′N, 20°33′E) and the Institute of field and vegetable crops in Novi Sad (45°33′N, 19°84′E), Serbia, during the winter and summer seasons of 2015. After field sampling, diapausing larvae were acclimated to 5 °C for two weeks in controlled laboratory conditions and then gradually chilled by lowering the temperature by 1 °C each day (Fig. 6). After the two-week cold acclimation at specific temperature points (5 °C, −3 °C and −16 °C), larvae were frozen in liquid nitrogen and then stored at −80 °C until analysis. Non-diapausing larvae and pupae were collected during June and July of 2015 and were also frozen in liquid nitrogen and stored at −80 °C until analysis.

Overall, five experimental groups were established (Fig. 6) – three cold acclimated groups of diapausing 5th instar larvae: *D(5)* – acclimated to 5 °C; *D(−3)* – acclimated to −3 °C and *D(−16)* – acclimated to −16 °C; and two actively developing groups: *ND* – 5^th^ instar non-diapausing larvae (control) and *P* – pupae. Each experimental group was comprised of 5 biological replicates (pools), with each pool consisting of 10 randomly chosen larvae or pupae.

### Tissue preparation

Whole larvae and pupae were homogenized in ice-cold 50 mM phosphate buffer pH 7 (20% w/v homogenates). To remove insoluble debris, the crude homogenates were first centrifuged for 10 minutes at 1000 g and 4 °C. Then, the supernatants were transferred to new microtubes and centrifuged again for 10 minutes at 5000 g and 4 °C, to remove lipid leftovers. Total proteins were measured in the purified supernatants on a 250 µl microplate assay using a commercial Bradford reagent kit (Quick Start™ Bradford Protein Assay), according to the manufacturer’s instructions (Bio-Rad, cat. no. 5000203).

### Enzyme activity analyses

The activity of CS was measured by a continual spectroscopic method according to Srere^67^. The basic CS enzyme reaction was coupled with an indicator reaction in which dinitrobenzoic acid (DTNB) is converted into 2-nitro-5-tiobenzoate (TNB), with an absorption peak at 412 nm. CS activity was determined by measuring the linear increase of absorbance of TNB within the first 3 minutes of the reaction.

The spectrophotometric assay for LDH activity estimation was performed according to a modified method by Henry *et al*.^68^, using pyruvate as a substrate and NADH as a coenzyme. LDH activity was determined by measuring the linear decrease of NADH absorbance at 340 nm within the first 6 minutes of the reaction. In order to identify the presence of the different LDH isoforms and also to measure total LDH activity in the opposite direction – towards pyruvate production, LDH isoenzymes were separated from whole-body homogenates with native disc PAGE (30 μg of total proteins per well), while in-gel LDH activity was detected using Na-lactate as substrate and nitroblue tetrazolium (NBT) as the chromogenic compound^69,70^. Gels were documented using BioDocAnalyze (BDA) digital system (Biometra, Germany) and the relative abundance of the bands was quantified in the program ImageJ (Rasband, W.S., ImageJ, U. S. National Institutes of Health, Bethesda, Maryland, USA, https://imagej.nih.gov/ij/, 1997–2018).

The assay for the spectrophotometric determination of ALT and AST activity was performed according to a modified method by Henry *et al*.^68^. Measurements of ALT and AST activities were performed using commercial kits – Glutamate pyruvate transaminase Diagnostic Reagent Kit IFCC Method, Reanal Finechemical Co., cat. no. 11061-2-99-80 (for ALT) and Glutamate oxaloacetate transaminase Diagnostic Reagent Kit IFCC Method, Reanal Finechemical Co., cat. no. 11021-2-99-80 (for AST). Substrates for ALT and AST reactions were L-alanine and L-aspartate, respectively, and the resulting metabolites – pyruvate and oxaloacetate became the substrates for the corresponding reactions of lactate dehydrogenase and malat dehydrogenase, respectively, according to the manufacturer instructions. Finally, the activities of ALT and AST were proportional to the oxidation rate of NADH used in the corresponding indicator reactions and were determined by recording the linear decrease of absorbance at 340 nm within the first 3 minutes of measurement.

### Statistical tests

All enzyme assays were performed in technical duplicates for each of the five biological pools. Statistical analysis of the results was performed using the data analysis software system Statistica version 13 (StatSoft, Inc., Tulsa, OK, USA). No significant statistical difference was observed in the standard deviation among the technical duplicates. The statistical significance of the measurements was tested using one-way ANOVA followed by *post hoc* Fisher’s test, with a level of significance of p < 0,05. In addition, the data were analysed by Principal Component Analysis (PCA). The results are shown as univariate scatterplots, as recommended for small sample size studies^71^.

## Supplementary information

Supplementary information.

## Data Availability

The datasets generated and analysed during the current study are available from the corresponding author on reasonable request.
