# Supplementary material for: Effect of Cold Acclimation on Selected Metabolic Enzymes During Diapause in The European Corn Borer Ostrinia nubilalis (Hbn.)
Source: Sci Rep. 2020 Jun 3;10:9085. doi: 10.1038/s41598-020-65926-w (PMC7270089; doi:10.1038/s41598-020-65926-w)
Supplement: Supplementary file 1 — Supplementary information. [file 41598_2020_65926_MOESM1_ESM.pdf]

EFFECT OF COLD ACCLIMATION ON SELECTED METABOLIC ENZYMES DURING DIAPAUSE IN THE EUROPEAN CORN BORER *OSTRINIA NUBILALIS* (HBN.)

Iva Uzelac[1], Miloš Avramov[1], Tatjana Čelić[1], Elvira Vukašinović[1], Snežana Gošić-Dondo[2], Jelena Purać[1], Danijela Kojić[1], Duško Blagojević[3], Željko D. Popović[1]*

[1]University of Novi Sad, Faculty of Sciences, Department of Biology and Ecology,

Trg Dositeja Obradovića 2, 21000 Novi Sad, Serbia

[2]Maize Research Institute, Zemun Polje, Slobodana Bajića 1, 11185 Belgrade, Serbia

[3]Institute for Biological Research "Siniša Stanković", Bulevar despota Stefana 142,

11060 Belgrade, Serbia

*Corresponding author: Željko D. Popović, Associate Professor

tel. +38121/485-2797, fax +38121/450-620

e-mail: zeljko.popovic@dbe.uns.ac.rs

Full-length gels that were used in the making of Fig. 2 and Fig. 3 in the Article are displayed in this Supplementary file. Gels were documented using BioDocAnalyze (BDA) digital system (Biometra, Germany). Lanes belonging to each biological pool are designated with brackets and pool symbols (D(5), diapausing larvae acclimated at 5 °C; D(-3), diapausing larvae acclimated at -3 °C; D(-16) diapausing larvae acclimated at -16 °C; ND—non-diapause and P—pupae).

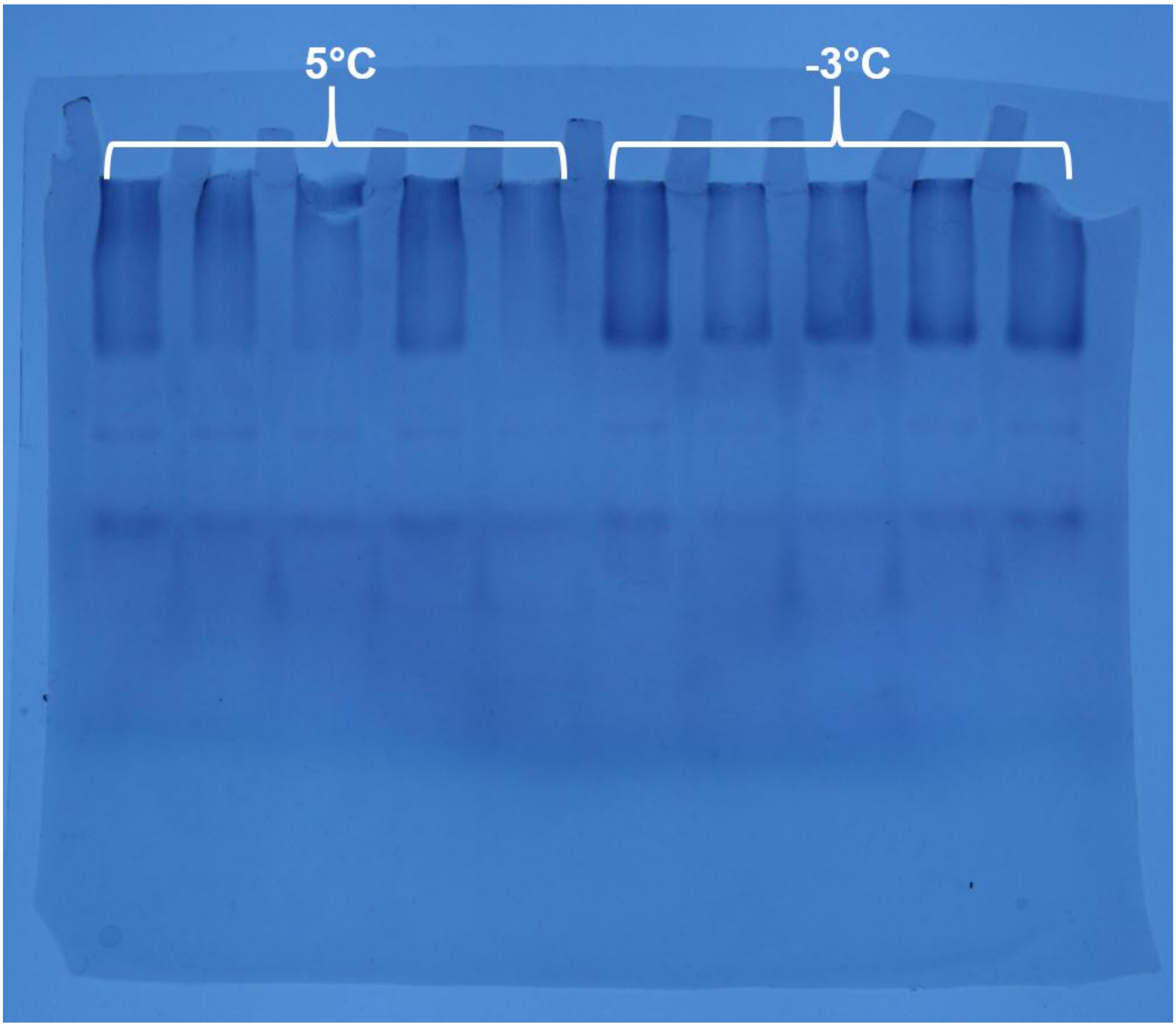

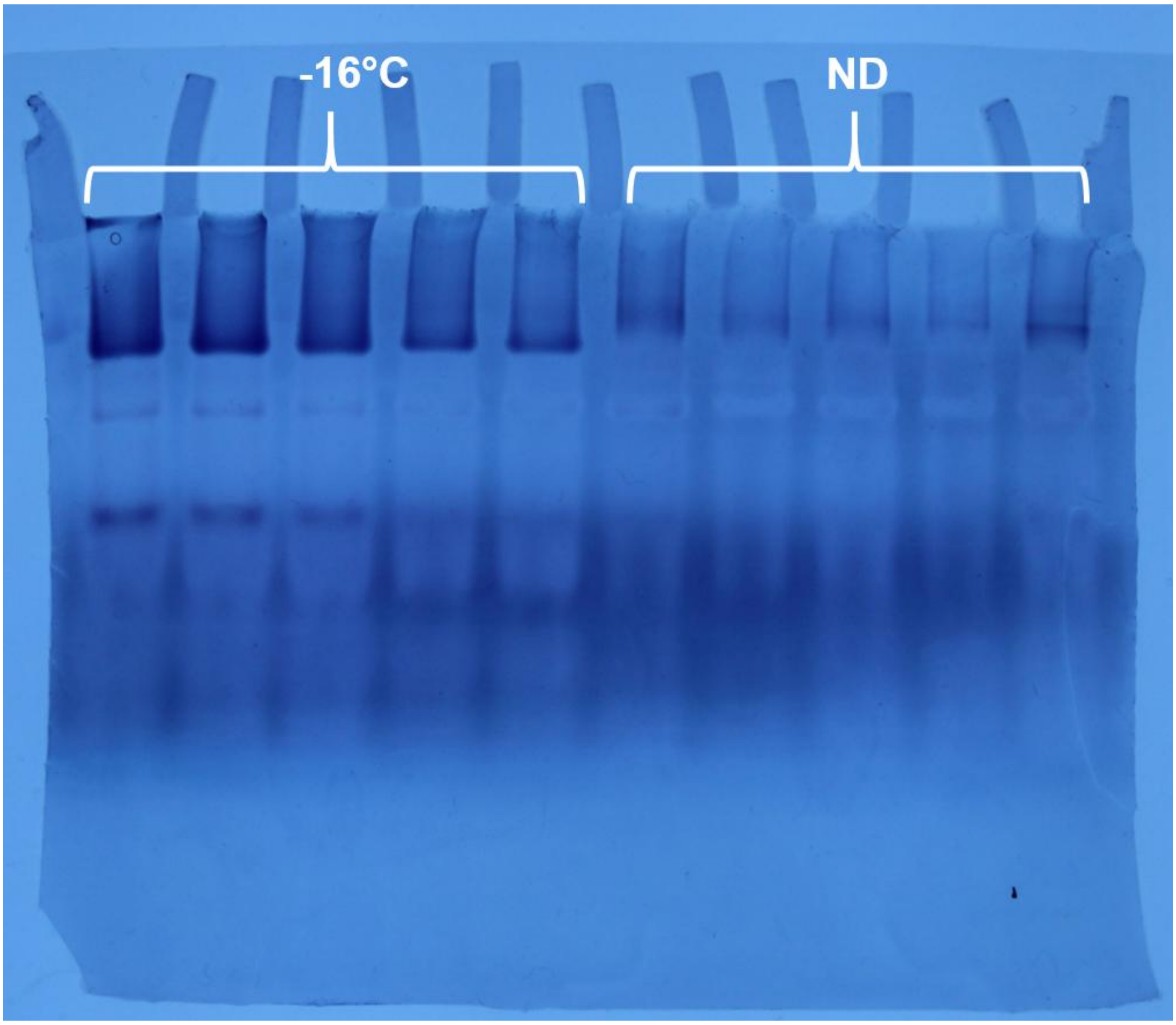
-16°C
ND

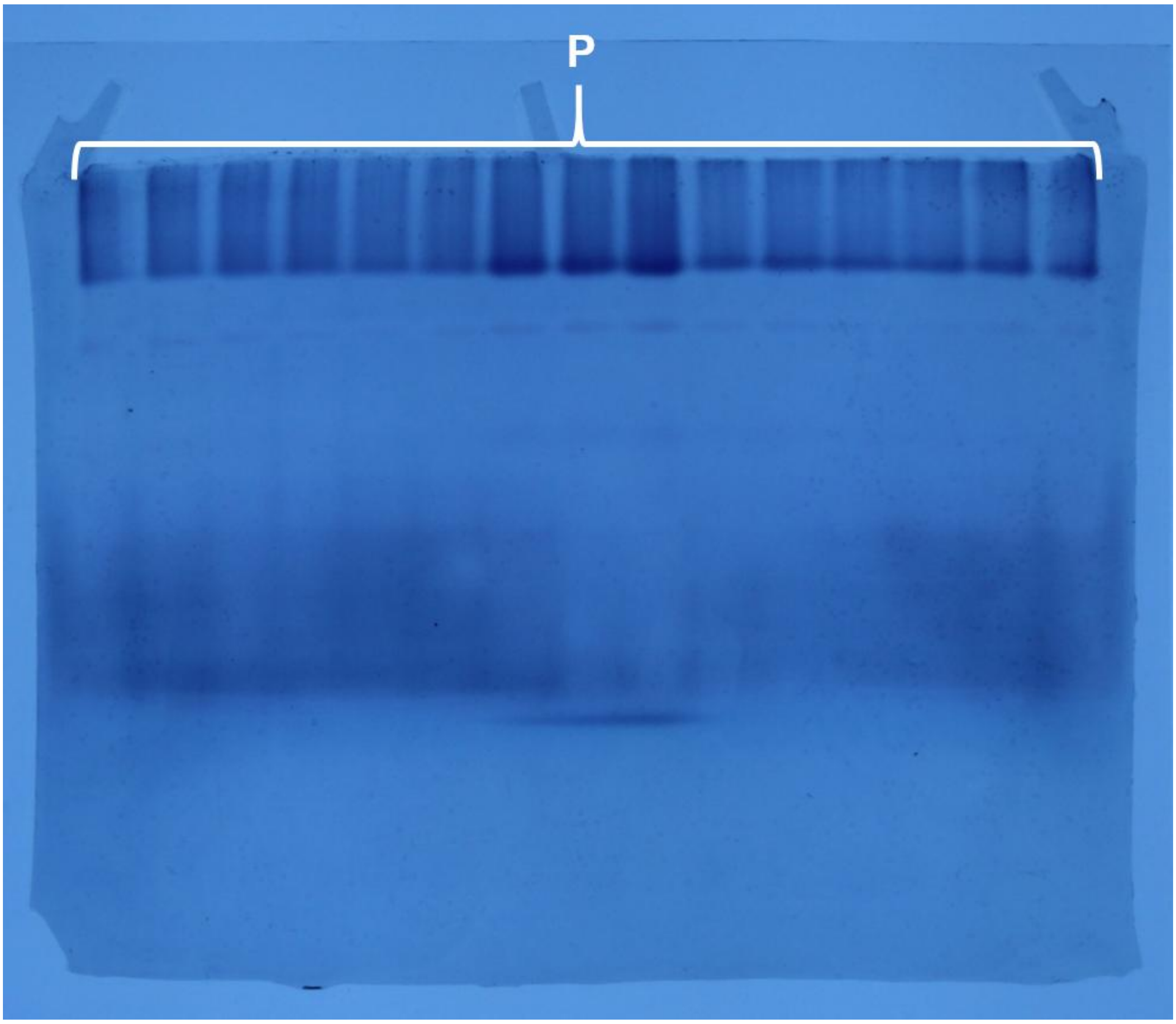
P
